# Impact of NK Cell Activating Receptor Gene Variants on Receptor Expression and Outcome of Immunotherapy in Acute Myeloid Leukemia

**DOI:** 10.3389/fimmu.2021.796072

**Published:** 2021-12-09

**Authors:** Brwa Ali Hussein, Alexander Hallner, Lovisa Wennström, Mats Brune, Anna Martner, Kristoffer Hellstrand, Elin Bernson, Fredrik B. Thorén

**Affiliations:** ^1^ Tumor Immunology (TIMM) Laboratory at Sahlgrenska Center for Cancer Research, University of Gothenburg, Gothenburg, Sweden; ^2^ Department of Medical Biochemistry and Cell Biology, Institute of Biomedicine, University of Gothenburg, Gothenburg, Sweden; ^3^ Department of Infectious Diseases, Institute of Biomedicine, University of Gothenburg, Gothenburg, Sweden; ^4^ Department of Hematology, Institute of Medicine, University of Gothenburg, Gothenburg, Sweden; ^5^ Department of Obstetrics and Gynecology, Institute of Clinical Sciences, University of Gothenburg, Gothenburg, Sweden

**Keywords:** NK cell receptors, gene variants, single nucleotide polymorphism, acute myeloid leukemia, Histamine/IL-2, Re:Mission trial, immunotherapy

## Abstract

Natural killer cells are important effector cells in the immune response against myeloid malignancies. Previous studies show that the expression of activating NK cell receptors is pivotal for efficient recognition of blasts from patients with acute myeloid leukemia (AML) and that high expression levels impact favorably on patient survival. This study investigated the potential impact of activating receptor gene variants on NK cell receptor expression and survival in a cohort of AML patients receiving relapse-preventive immunotherapy with histamine dihydrochloride and low-dose IL-2 (HDC/IL-2). Patients harboring the G allele of rs1049174 in the *KLRK1* gene encoding NKG2D showed high expression of NKG2D by CD56^bright^ NK cells and a favorable clinical outcome in terms of overall survival. For DNAM-1, high therapy-induced receptor expression entailed improved survival, while patients with high DNAM-1 expression before immunotherapy associated with unfavorable clinical outcome. The previously reported SNPs in *NCR3* encoding NKp30, which purportedly influence mRNA splicing into isoforms with discrete functions, did not affect outcome in this study. Our results imply that variations in genes encoding activating NK cell receptors determine receptor expression and clinical outcome in AML immunotherapy.

## Introduction

Acute myeloid leukemia (AML) is a hematopoietic malignancy characterized by uncontrolled expansion of myeloid progenitors at the expense of normal hematopoiesis. AML is the most common form of acute leukemia in adults and is associated with poor long-term survival (1, 2). Upon diagnosis, patients undergo induction chemotherapy, which mostly results in the disappearance of morphologically detectable leukemia along with the return of normal hematopoiesis (complete remission, CR). To prevent relapse, patients subsequently receive additional rounds of consolidation chemotherapy. Eligible patients with intermediate or high-risk AML may undergo allogeneic stem cell transplantation (allo-SCT). Relapses are however common in the post-consolidation phase, and there remains a need for additional post-consolidation therapy, in particular for non-transplanted patients (3–5).

The last decade has seen a paradigm shift in AML treatment towards targeted therapies along with the introduction of immunotherapeutic strategies. Gene mutation-targeted agents like FLT3 and IDH inhibitors have shown promise in AML (6, 7), and a combinatory immunotherapy comprising histamine dihydrochloride and interleukin-2 (HDC/IL-2) is approved for relapse prevention for AML in the European Union (8). HDC blocks the formation of immunosuppressive reactive oxygen species (ROS) from normal and malignant myeloid cells, while IL-2 stimulates proliferation and activation of natural killer (NK) cells and T cells (9–12).

NK cells are cytotoxic lymphocytes that are regulated by inhibitory and activating receptors. The main inhibitory receptors recognize HLA molecules expressed at high levels by most nucleated cells. Downregulation of HLA, which may be utilized by malignant cells to evade T cell recognition, allows activation of NK cells in a process known as missing self-recognition (13). Among the broad range of activating receptors on NK cells are the family of natural cytotoxicity receptors (NCRs), *i.e.*, NKp30, NKp44 and NKp46; NKG2D, DNAM-1 and CD16 (14, 15). In AML, reduced expression of NK cell NCRs is associated with poor leukemia-free survival (LFS) and overall survival (OS) (16, 17), thus highlighting the role of NK cells in controlling leukemic cells in AML.

NK cell receptor expression is regulated at multiple levels. Immunosuppressive mediators, such as ROS and TGF-beta, reportedly downregulate activating NK cell receptors (18, 19), and chronic engagement of activating receptors by cellular, humoral or exosome-associated ligands may trigger receptor internalization, degradation or desensitization (20–22). Furthermore, single nucleotide polymorphisms (SNPs) in coding or regulatory regions of NK cell receptor genes may result in enhanced or reduced expression, and gene variants may entail reduced affinity between receptors and their cognate ligands. Several SNPs in NK receptor genes have been associated with cancer risk and progression. A haplotype in the NK gene complex (NKC) locus, which encodes NK cell receptors such as NKG2D, NKG2A and NKG2C, was reported to be associated with a high-cytotoxicity profile and low cancer incidence (23). Espinoza et al. reported an association between NKG2D rs1049174 gene variants, located in the 3`-untranslated region of NKG2D gene, and the cytotoxic activity of NK cells and risk of developing cancer (24). The NKG2D rs1049174 is located in the binding site of microRNA-1245, which negatively regulates the expression of the NKG2D receptor. Thus, the NKG2D allele with a higher affinity to microRNA-1245 was found to be associated with low NKG2D expression and low natural cytotoxicity (24).

DNAM-1 is a key activating NK cell receptor, and variation at SNP rs763361 (C/T) within the gene encoding DNAM-1 results in glycine or serine at position 307 (Gly307Ser) (25). The T allele at rs763361 is associated with increased risk of type 1 diabetes, systemic sclerosis and systemic lupus erythematosus (26). Additionally, the TT genotype of rs763361 is common in patients with non-small cell lung cancer (27). The expression of DNAM-1 by NK cells has been reported to be reduced in AML with ensuing inefficiency of NK cell-mediated elimination of leukemic cells (28), but the impact of DNAM-1 gene variation on receptor expression, downstream signaling and effector functions of NK cells is incompletely understood.

In the gene encoding NKp30, a combination of SNPs reportedly associates with changes in the ratio of splice variants, which in turn impacts on the effector functions of NKp30 ligation. Downstream signaling from NKp30 isoforms A/B mediates cytotoxic responses and IFN-γ production, whereas the NKp30C isoform leads to the production of IL-10 (29). Notably, the NKp30C isoform was reported to negatively impact the prognosis of patients with melanoma and metastatic gastrointestinal stromal tumors (GIST) (29, 30).

These previous studies incited us to investigate to what extent genetic variation at SNPs that encode activating NK cell receptors, including NKG2D, DNAM-1 and NKp30, affect expression levels and outcome in AML patients receiving NK cell-activating immunotherapy. Our findings denote that NK cell expression of activating receptors is associated with clinical outcome of immunotherapy. We also displayed gene variants that may determine NK cell receptor expression of relevance to patient survival.

## Patients and Methods

### Patients

Eighty-four patients aged 18 years or older (18-79) with *de novo* or secondary AML in their first complete remission after receiving induction and consolidation chemotherapy were enrolled in a single-armed multicenter phase IV clinical trial (ReMission, NCT01347996). Details regarding induction and consolidation therapy can be found in (31). Three patients were excluded from the study after they withdrew the consent. The total number of a combination maintenance treatment of histamine dihydrochloride and IL-2 included 10 consecutive 3-week cycles of histamine dihydrochloride (HDC; 0.5 mg, subcutaneously twice daily) and low-dose IL-2 (16 400 IU/kg, subcutaneously twice daily) for 18 months or until disease relapse or death. Patients had 3-week off treatment in between cycles 1-3 and 6-week off treatment in cycles 4-10. The primary end points of the study involved assessment of both quantitative and qualitative pharmacodynamic impact of HDC/IL-2 on immunological response related to NK cell and T cell biology, and results for this phase IV clinical trial’s primary end points can be found in (16, 32–34), with patient characteristics published in (16). The trial was approved by ethics committees from each participating institution and was conducted according to the principles of the Declaration of Helsinki. Written informed consent before enrollment in the study was obtained from each study participant.

### Peripheral Blood Sampling and Flow Cytometry Analysis

Peripheral blood was collected from patients before and after first and third HDC/IL-2 treatment cycles. PBMCs were isolated and cryopreserved at local sites and then shipped to TIMM laboratory on dry ice and stored at -180°C. Later, PBMCs from before and after first treatment cycle were thawed and analyzed by flow cytometry to assess expression of NK activating receptors including NKG2D, DNAM-1 and NKp30 (expression presented in median fluorescence intensity; MFI). Cells were stained with LIVE/DEAD Fixable Yellow or NearIR Dead Stain kit (Invitrogen). After washing, cells were stained with anti-CD3-FITC (HIT3a), CD3-APC-H7 (SK7), CD16-V450 (3G8), CD16-AlexaFluor700 (3G8), CD56-APC (B159), CD56-BV711 (NCAM-1), CD14-APC-H7 (MpP9); all BD Biosciences), CD19-APC-H7 (SJ25C1; BD Pharmingen), anti-NKp30-PE (AF29-4D12), NKG2D-biotin (BAT221), DNAM-1-PE-Vio770 (DX11; all Miltenyi Biotech) and Streptavidin-Odot605 (Invitrogen). Samples were collected on a 4-laser BD LSRFortessa SORP (BD Biosciences) and data were analyzed with FACSDiva software version 6 or later, or FlowJo software version 8 or later. The gating strategy of flow cytometry data is depicted in **Supplemental Figure 1**. In brief, cells, single cells and lymphocytes were gated based on scatter characteristics, and dead cells together with T-cells, B-cells and monocytes were removed using a dump channel (CD3, CD14, CD19, and a live dead marker). NK cells were identified as dump-negative and CD16^-^CD56^bright^ or CD16^+^CD56^+^. Median fluorescence intensity (MFI) for NKG2D, DNAM-1 and NKp30 was evaluated for each NK cell subset.

### Genotyping of NKp30, DNAM-1 and NKG2D Single Nucleotide Polymorphisms

Genomic DNA samples were isolated from PBMCs of patients enrolled in the ReMission trial using the Qiagen^®^ DNeasy Blood & Tissue kit. Four NK cell activating receptor gene variants including NKp30 (rs986475), NKp30 (rs1052248), DNAM-1 (rs763361) and NKG2D (rs1049174) were genotyped using the TaqMan-Allelic discrimination with Applied Biosystem 7500 Fast Real-Time PCR System. All predesigned TaqMan SNP genotyping assays were ordered from Thermo Fisher Scientific.

### Statistical Analysis

Statistical analysis was performed using GraphPad prism version 9.1.0. Unpaired and paired two-sided Student’s *t*-test was applied to compare two groups. Linear regression analysis was performed to determine the correlation between gene variant alleles and receptor expression. Overall survival (OS) and leukemia-free survival (LFS) analyses were executed using the log rank test. Impact of age on survival was further analyzed using multivariate cox regression analysis.

## Results

### An NKG2D Gene Variant Impacts on the Outcome of Immunotherapy in AML

**Figure 1:**
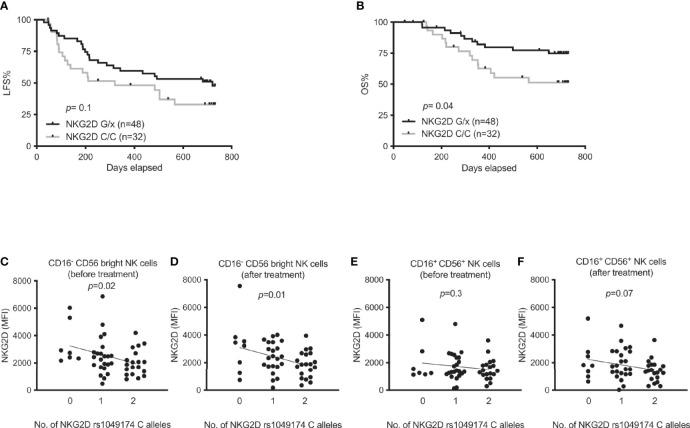
Impact of NKG2D rs1049174 gene variants on outcome of immunotherapy in AML and expression of NKG2D. **(A, B)** Leukemia-free survival (LFS) and overall survival (OS) of AML patients harboring NKG2D G/x (n=48) or CC (n=32) after receiving HDC/IL-2 treatment. **(C–F)** NKG2D expression according to NKG2D rs1049174 genotypes in AML patients in both CD16^-^ CD56^bright^ and CD16^+^CD56^+^ NK cells before and after immunotherapy as indicated in figures. Patients in figures **(C–F)** are divided according to indicated number of NKG2D rs1049174 C alleles [n equals 7, 23 and 19 before immunotherapy; panels **(C, E)**; and 8, 23 and 20 after immunotherapy; panels **(D, F)**]. *P* values were obtained using log rank test for survival in figures **(A, B)** and simple linear regression analysis was performed in figures **(C–F)**.

Several single nucleotide polymorphisms in the NKC gene locus affect NK cell responses to malignant cells and may be associated with cancer incidence. Most of these SNPs are located in the vicinity of the *KLRK1* gene that encodes the activating NK cell receptor NKG2D (23). To determine the impact of NKG2D on AML immunotherapy, we genotyped AML patients from the Re:Mission trial for the rs1049174 SNP. As shown in **Figures 1A, B**, patients with at least one G allele showed significantly higher overall survival compared with patients carrying the CC genotype (p=0.04) with a similar trend for leukemia-free survival. To identify the mechanisms of relevance to the superior OS of rs1049174 G/x patients, we determined the expression levels of NKG2D in trial patients. NKG2D expression was not significantly induced on NK cells following immunotherapy (data not shown), but a linear regression analysis showed that expression of NKG2D was inversely correlated with the number of C alleles at rs1049174 in the immature CD16^-^ CD56^bright^ NK cell subset at onset (p= 0.02) and after the first cycle (p=0.01) of HDC/IL-2 immunotherapy (**Figures 1C, D**). A similar albeit non-significant trend was noticed for the more cytotoxic CD16^+^CD56^+^ NK cell subset after treatment (**Figures 1E, F**). In line with these findings, above-median expression of NKG2D by the CD16^-^ CD56^bright^ subset after one cycle of therapy was associated with a trend towards impact on LFS and OS, whereas no effect on survival was observed when patients were dichotomized based on above- or below-median expression of NKG2D in the CD16^+^CD56^+^ NK cell subset (**Supplemental Figures 2A-D**).

### A DNAM-1 Gene Variant Correlates With Receptor Expression in AML Patients

**Figure 2:**
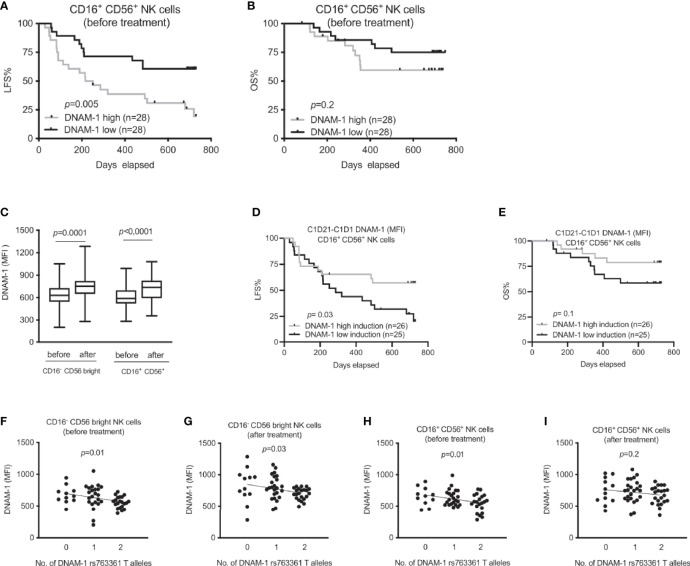
Association between DNAM-1 expression and outcome of immunotherapy in AML. **(A, B)** Leukemia-free survival (LFS) and overall survival (OS) of AML patients before immunotherapy according to DNAM-1 expression (MFI) in specified NK cell subsets. **(C)** Box plot shows effect of HDC/IL-2 therapy on median fluorescence intensity (MFI) of DNAM-1 expression in both CD16^-^ CD56^bright^ and CD16^+^ CD56^+^ NK cells of AML patients both at the beginning (n=51) and after (n=59) of treatment. **(D, E)** Impact of induction of the CD16^+^ CD56^+^ NK DNAM-1 expression on LFS and OS during first three weeks of immunotherapy in AML. Patients were dichotomized based on above (grey) and below (black) median expression (MFI) of DNAM-1. **(F–I)** Expression of DNAM-1 in AML patients during immunotherapy based on number of DNAM-1 rs763361 T alleles in CD16^-^CD56^bright^ and CD16^+^ CD56^+^ NK cells before and after receiving one cycle of HDC/IL-2 therapy. Patients are divided according to indicated number of T alleles [n equals 11, 23 and 21 before immunotherapy; panels **(F, H)**; and 12, 24 and 22 after one cycle of immunotherapy; panels **(G, I)**]. Paired two-sided *t* test was performed to analyze the difference in DNAM-1 expression before and after therapy in different NK cell subsets in figure **(C)**. Logrank test was carried out for survival analysis in figures **(A, B, D, E)**. Simple linear regression was implemented to investigate impact of DNAM-1 rs763361 gene variants on expression of DNAM-1 both before and after immunotherapy in various NK subsets as shown in figures **(F–I)**.

NK cells from AML patients younger than 65 years showed reduced expression of DNAM-1 when compared to healthy controls (28). Thus, we next investigated how DNAM-1 expression before and after first treatment cycle impacted on survival in this cohort of AML patients receiving HDC/IL-2 immunotherapy. Surprisingly, our analysis showed that a below median DNAM-1 expression correlated with an increased leukemia-free survival before treatment start, while the impact of DNAM-1 expression on overall survival before treatment did not reach significance (**Figures 2A, B**). This finding was not due to differential DNAM-1 expression among young and elderly patients, as low DNAM-1 expression remained a significant predictor of LFS in a multivariate analysis correcting for age (**Supplemental Table 1**). By contrast, DNAM-1 expression after treatment did not impact on leukemia-free survival and overall survival (data not shown). However, we noted that CD16^-^CD56^bright^ as well as CD16^+^CD56^+^ cells showed increased expression of DNAM-1 after the first cycle of HDC/IL-2 (**Figure 2C**), and patients with above median increase in fluorescence intensity (MFI) values between treatment start and three weeks of treatment showed superior leukemia-free survival as compared with patients with low or no increase in DNAM-1 expression (**Figures 2D, E** and **Supplemental Table 1**; log rank test, p=0.03 and p=0.1 for LFS and OS, respectively). Our data showing that high induction of DNAM-1 was protective in the trial while high DNAM-1 expression did not impact on clinical outcome, made us widen our investigation of the impact of DNAM-1 by studying how the genetic DNAM-1 variant rs763361 (T or C) impacted on receptor expression and treatment outcome in this AML patient cohort. The rs763361 T gene variants in the gene encoding DNAM-1 have been suggested to predispose to diabetes and SLE (26), indicating that this SNP may impact on DNAM-1 expression or function. Linear regression analysis of DNAM-1 expression and number of T alleles at rs763361 revealed that the T allele was associated with low DNAM-1 expression (**Figures 2F–I**); however, the rs763361 DNAM-1 SNP did not affect treatment outcome in this trial (**Supplemental Figures 3A–B**).

### NKp30 Gene Variants Differentially Affect NKp30 Expression in AML Patients

**Figure 3:**
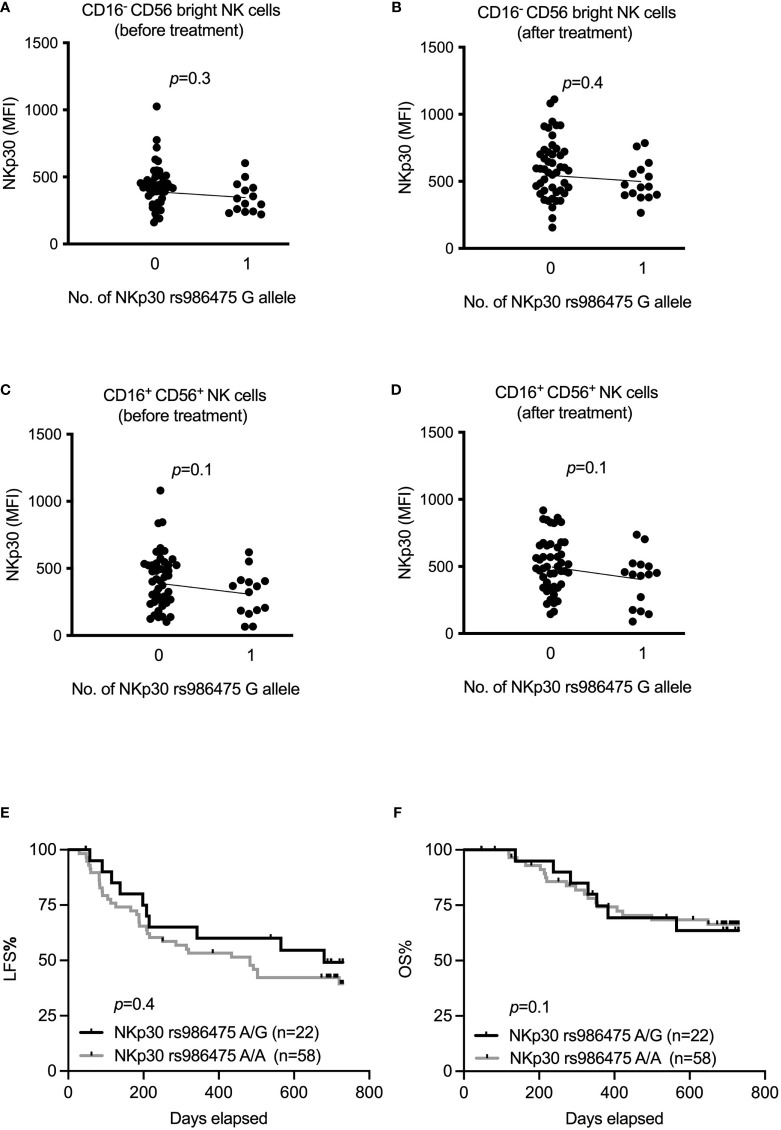
Impact of NKp30 rs986475 gene polymorphism on expression of NKp30 and clinical outcome of AML during immunotherapy. **(A–D)** Median fluorescence intensity of NKp30 based on genotype status of NKp30 rs986475 in AML patients in both CD16^-^ CD56^bright^ and CD16^+^ CD56^+^ NK cells before and after receiving one cycle of HDC/IL-2 therapy. Patients are divided according to presence (14 out of 61 before therapy, and 15 out of 62 after therapy) or absence of G allele in figures **(A–D)**. **(E, F)** Kaplan-Meier curves show impact of different NKp30 rs986475 genotypes on leukemia-free survival (LFS) and overall survival (OS) of AML patients after receiving HDC/IL-2 therapy. Simple linear regression was applied to investigate impact of NKp30 gene variants on NKp30 expression both before and after immunotherapy in various NK subsets as shown in figures **(A–D)**. Logrank test was used to analyze the survival based on NKp30 gene variants in figures **(E, F)**.

In previous studies, we have reported that AML patients strongly upregulate expression of NKp30 in response to HDC/IL-2 immunotherapy (16), and that high NKp30 expression is associated with improved LFS and OS in elderly AML patients (35). In a set of papers, Zitvogel and coworkers reported that NKp30 gene variants generate different ratios of splice variants that affect the functional outcome of NKp30 ligation (29, 30). To address whether these variants are of relevance in AML immunotherapy, we determined the genotype of two major NKp30 gene variants, rs986475 (A or G) and rs1052248 (T or A) within the ReMission trial cohort. As shown in **Figures 3A–D**, our results only revealed a trend towards higher expression of NKp30 among patients carrying no G alleles at rs986475 in the CD16^-^ CD56^bright^ and CD16^+^ CD56^+^ subsets before or after treatment. We investigated whether the rs986475 variant correlated with clinical outcome in the ReMission trial, but did not observe any impact of the different gene variants on LFS or OS (**Figures 3E, F**). The gene variants at rs1052248 did not entail differences in NKp30 expression in any NK subset or at any treatment time point (**Supplemental Figures 4A–D**). Moreover, there was no correlation between clinical outcome and gene variants of rs1052248 (**Supplemental Figures 4E, F**). In their investigation on how NKp30 gene variants affect GIST outcome, the Zitvogel team grouped patients based on a combination of rs986475 and rs1052248, as a model predicting the expression of an immunosuppressive NKp30 isoform. Thus, we investigated whether combinations of the two SNPs impacted on clinical outcome in our cohort of AML patients, but no combination resulted in a significant difference in clinical outcome (data not shown).

## Discussion

Variants of genes encoding activating NK cell receptors have been reported to impact on NK cell function and cancer incidence as well as on outcome in diseases such as GIST and SLE (26, 29). In this study, we investigated how gene variants of the activating NK cell receptors including NKG2D, DNAM-1 and NKp30 impact on clinical outcome and receptor expression in a cohort of AML patients receiving IL-2-based immunotherapy.

Imai et al. showed that individuals displaying low natural cytotoxicity were at increased cancer risk 11 years later (36). In a follow-up study, these authors found a haplotype in the NKC region that was associated with high NK cell cytotoxicity and reduced cancer incidence. The majority of SNPs associated with altered NK cell cytotoxicity were found in the *KLRK1* gene encoding NKG2D (23). Furthermore, one study reported an association between the G allele of NKG2D of donors and the clinical outcome of hematopoietic stem cell transplantation in patients with standard risk hematological malignancies (37). In accordance with these studies, we observed significantly higher overall survival for patients with at least one G allele in rs1049174 with a similar trend for leukemia-free survival. NKG2D expression was also enhanced on CD56^bright^ cells in these patients, while the effect was more modest in the mature CD16^+^CD56^+^ compartment. While it is conceivable that high expression of an activating NK cell receptor is associated with improved survival, it should be emphasized that there is considerable linkage disequilibrium between different SNPs in the NKC locus, and the locus harbors several genes that encode receptors with impact on NK cell function, including NKp80, NKG2A and NKG2C. The association between the SNP, rs1049174, and survival after AML immunotherapy may thus not necessarily reflect effects on NKG2D expression. For example, the rs1049174 dimorphism is associated with induced NKG2A expression in this cohort (data not shown), and additional studies are needed to tease out the consequences of these differences in expression levels among patients.

The DNAM-1 receptor and its inhibitory counterparts, TIGIT and Tactile, have been implicated in cancer of multiple histotypes (28, 38–40). In this study, we observed a robust induction of DNAM-1 expression after HDC/IL-2 immunotherapy, and patients with above-median induction of DNAM-1 expression between start and the first three weeks of treatment had superior LFS and OS. The DNAM-1 rs763361 T allele, which has been implicated in autoimmunity and cancer (26, 27) was associated with lower DNAM-1 expression in both major NK cell subsets but did not impact on LFS or OS in our cohort of patients. Paradoxically, we found that high expression of DNAM-1 before treatment start was associated with reduced LFS. Notably, DNAM-1 expression has been reported to be associated with NK cell education (41, 42). Thus, DNAM-1 expression is higher in NK cells that express at least one killer immunoglobulin-like receptor (KIR), that recognizes a self HLA molecule. We have previously reported that patients with an HLA genotype lacking one of the HLA molecules that serve as KIR ligands show superior survival, which may be related to the presence of an uneducated, autoreactive subset of NK cells in these patients (32). It is thus possible that the enhanced survival of patients with low DNAM-1 expression before treatment start is related to, at least in part, a lower education level and presence of autoreactive NK cells in this subset of patients. It should also be emphasized that the improved survival observed in individuals with high increase in DNAM-1 expression during the first treatment cycle may merely reflect a benefit of a general treatment response in terms of NK cell activation rather than an effect exerted by the receptor DNAM-1 itself.

Three different isoforms of NKp30 have been described, two immunostimulatory and one immunosuppressive, as a result from alternate splicing of the *NCR3* gene (29). In a cohort of gastrointestinal stromal tumor patients, expression of the immunosuppressive NKp30 isoform was associated with poor survival (29). A SNP at position 3790 in the 3’ untranslated region of the gene encoding NKp30 was reported to partly explain the expression of the immunosuppressive NKp30 isoform, where presence of rs986475 G associated with the immunosuppressive NKp30 isoform (29). Moreover, absence of the rs986475 G allele has been linked to long survival in melanoma patients (30). The impact of NKp30 on outcome in cancer is not only driven by differential expression of NKp30 isoforms. A high expression of NKp30 may improve NK cell recognition of malignant cells, and accordingly, we found high NKp30 expression to be strongly protective during IL-2-based immunotherapy in elderly patients with AML (16). In this study, we detected a trend towards higher NKp30 expression in patients homozygous for the purported protective variant at rs986475, but this did not translate into favorable clinical outcome in our AML patient cohort. The other NCR3 gene polymorphism (rs1052248) associated with the NKp30 immunosuppressive isoform did not affect survival, nor did any combination of these SNPs. A potential limitation of our study is that we did not have the opportunity to address to what extent the NCR3 genotypes affected the relative abundance of the different NKp30 isoforms expressed by the patients’ NK cells. However, the previous studies reporting differences in outcomes due to NKp30 isoforms did observe effects when solely taking SNP data into consideration. Taken together, we did not find any impact of NKp30 gene variants on survival in AML patients receiving HDC/IL-2 immunotherapy.

In conclusion, our results show that the SNP rs1049174 in the gene encoding NKG2D, is associated with both NKG2D expression level and clinical outcome for AML patients receiving IL-2 based immunotherapy. In contrast, we did not observe an association between SNPs in genes encoding the activating NK cell receptors DNAM-1 and NKp30 and clinical outcome in this AML patient cohort. Our study suggests that genetic variations of activating NK cell receptor genes impact on receptor expression and function, which may determine the survival of AML patients receiving immunotherapy.

## Data Availability Statement

The original contributions presented in the study are included in the article/**Supplementary Material**. Further inquiries can be directed to the corresponding author.

## Ethics Statement

The trial (ReMission, NCT01347996) was approved by ethics committees from each participating institution and was conducted according to the principles of the Declaration of Helsinki. Written informed consent before enrolment in the study was obtained from each study participant. The patients/participants provided their written informed consent to participate in this study.

## Author Contributions

BH, EB, and FBT designed the study. BH performed experiments. BH, EB, and FBT analyzed research data. BH generated all figures and conceived first draft of manuscript. EB and FBT contributed to the writing and editing the text. All authors contributed to the article and approved the submitted version.

## Funding

This work was supported by the Swedish Research Council, the Swedish Cancer Society, the Swedish state *via* the ALF agreement, the Clas Groschinskys Foundation, the Åke Wiberg Foundation, the Assar Gabrielsson Foundation, the Lion Cancer Foundation West, the Wilhelm and Martina Lundgren Research Foundation, BioCARE, and the Sahlgrenska Academy at the University of Gothenburg.

## Conflict of Interest

Authors AM, KH, and FT are authors of issued or pending patents protecting the use of histamine dihydrochloride in cancer immunotherapy.

The remaining authors declare that the research was conducted in the absence of any commercial or financial relationships that could be construed as a potential conflict of interest.

## Publisher’s Note

All claims expressed in this article are solely those of the authors and do not necessarily represent those of their affiliated organizations, or those of the publisher, the editors and the reviewers. Any product that may be evaluated in this article, or claim that may be made by its manufacturer, is not guaranteed or endorsed by the publisher.
