# Corrigendum: Impact of NK Cell Activating Receptor Gene Variants on Receptor Expression and Outcome of Immunotherapy in Acute Myeloid Leukemia

**DOI:** 10.3389/fimmu.2021.843461

**Published:** 2022-01-13

**Authors:** Brwa Ali Hussein, Alexander Hallner, Lovisa Wennström, Mats Brune, Anna Martner, Kristoffer Hellstrand, Elin Bernson, Fredrik B. Thorén

**Affiliations:** ^1^ Tumor Immunology (TIMM) Laboratory at Sahlgrenska Center for Cancer Research, University of Gothenburg, Gothenburg, Sweden; ^2^ Department of Medical Biochemistry and Cell Biology, Institute of Biomedicine, University of Gothenburg, Gothenburg, Sweden; ^3^ Department of Infectious Diseases, Institute of Biomedicine, University of Gothenburg, Gothenburg, Sweden; ^4^ Department of Hematology, Institute of Medicine, University of Gothenburg, Gothenburg, Sweden; ^5^ Department of Obstetrics and Gynecology, Institute of Clinical Sciences, University of Gothenburg, Gothenburg, Sweden

**Keywords:** NK cell receptors, gene variants, single nucleotide polymorphism, acute myeloid leukemia, histamine/IL-2, Re:Mission trial, immunotherapy

In the original article, there was an error regarding **Figure 3D** as published. For purpose of clarification, AML patients enrolled in Re:Mission trial supposed to be divided into two groups based on NKp30 rs986475 gene variants (0=No G allele or 1=G allele carriers) in this **Figure 3D** but there is a third arm in the figure which is a patient with a zero value of NKp30 MFI and this was mistakenly added in GraphPad prism. The corrected version of **Figure 3** appears below.

**Figure 3:**
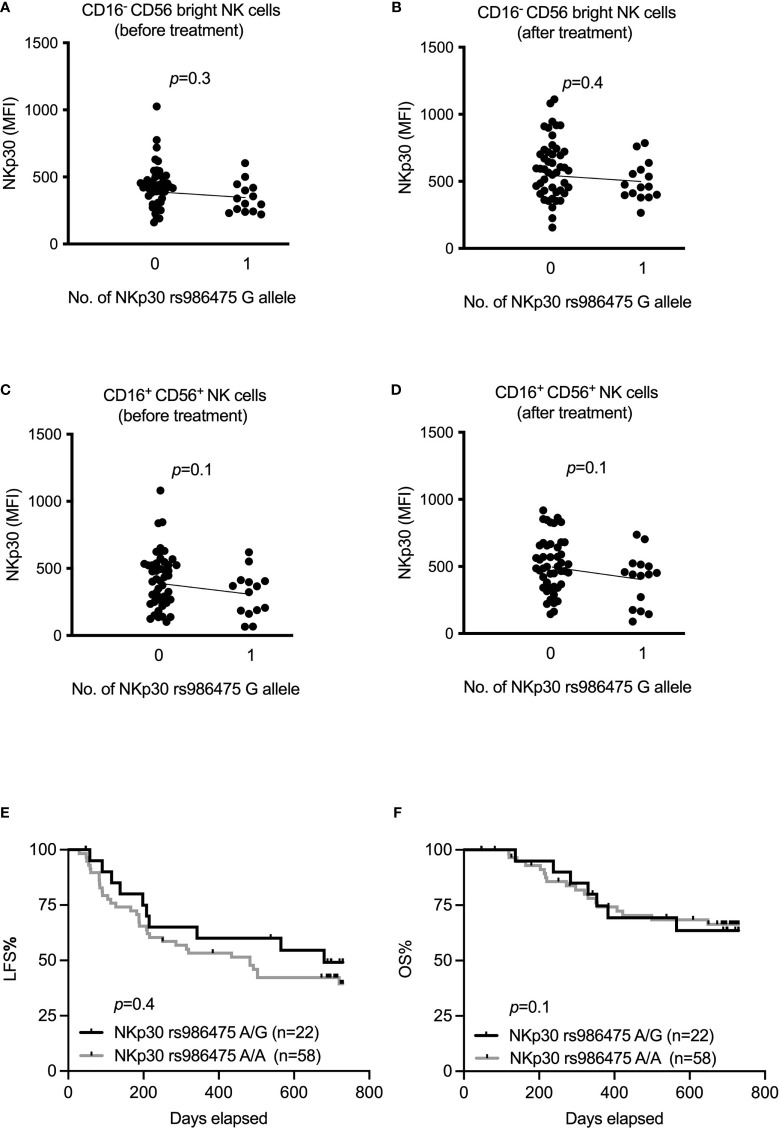
Impact of NKp30 rs986475 gene polymorphism on expression of NKp30 and clinical outcome of AML during immunotherapy. **(A–D)** Median fluorescence intensity of NKp30 based on genotype status of NKp30 rs986475 in AML patients in both CD16^-^ CD56^bright^ and CD16^+^ CD56^+^ NK cells before and after receiving one cycle of HDC/IL-2 therapy. Patients are divided according to presence (14 out of 61 before therapy, and 15 out of 62 after therapy) or absence of G allele in figures **(A–D)**. **(E, F)** Kaplan-Meier curves show impact of different NKp30 rs986475 genotypes on leukemia-free survival (LFS) and overall survival (OS) of AML patients after receiving HDC/IL-2 therapy. Simple linear regression was applied to investigate impact of NKp30 gene variants on NKp30 expression both before and after immunotherapy in various NK subsets as shown in figures **(A–D).** Logrank test was used to analyze the survival based on NKp30 gene variants in figures **(E, F)**.

The authors apologize for this error and state that this does not change the scientific conclusions of the article in any way. The original article has been updated.

## Publisher’s Note

All claims expressed in this article are solely those of the authors and do not necessarily represent those of their affiliated organizations, or those of the publisher, the editors and the reviewers. Any product that may be evaluated in this article, or claim that may be made by its manufacturer, is not guaranteed or endorsed by the publisher.

