# Supplementary material for: Impact of NK Cell Activating Receptor Gene Variants on Receptor Expression and Outcome of Immunotherapy in Acute Myeloid Leukemia
Source: Front Immunol. 2021 Dec 9;12:796072. doi: 10.3389/fimmu.2021.796072 (PMC8695486; doi:10.3389/fimmu.2021.796072)
Supplement: Supplementary file 1 [file DataSheet_1.docx]

**Supplementary materials**


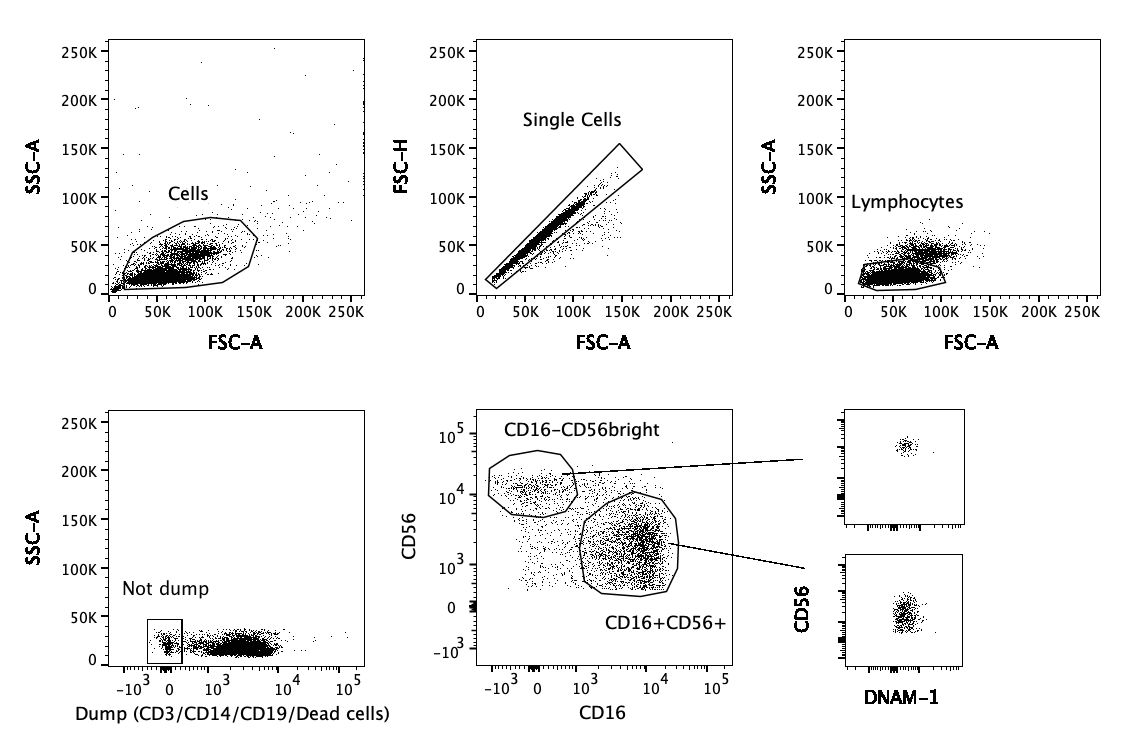


**Supplemental figure 1: Gating strategy of flow cytometry data.** FACS staining was performed in the following sequence: gating of cells, single cells and lymphocytes was implemented based on morphology in FSC/SSC plot. Fluorochromes including CD3, CD19, CD14, and live/dead marker were used in a dump channel to gate for T-cells, B-cells, monocytes, and dead cells. NK cells were identified as dump negative and CD16^-^CD56^bright^ or CD16^+^CD56^+^. Median fluorescence intensity (MFI) was calculated for each NK cell subset, as shown for DNAM-1 here.

**Supplemental figure 2: Impact of NKG2D expression on outcome of AML during immunotherapy.** **(A, B)** Leukemia-free survival (LFS) and overall survival (OS) of AML patients are plotted based on above (n=27) or below (n=26) median fluorescence intensity (MFI) of NKG2D expression on CD16^-^ CD56^bright^ NK cells after receiving one cycle of HDC/IL-2 therapy. **(C, D)** Leukemia-free survival and overall survival of AML patients according to above (n=31) or below (n=31) median expression of NKG2D of CD16^+^CD56^+^ NK cells after receiving one cycle of HDC/IL-2 therapy. *P* values were obtained using log rank test for survival in all figures.

**Supplemental figure 3: Correlation between expression of DNAM-1 and the clinical outcome of AML patients treated with IL-2 -based immunotherapy. (A, B)** Kaplan-Meier analysis of leukemia-free survival and overall survival for AML patients after receiving HDC/IL-2 therapy according to DNAM-1 genotype status; CC (n = 17), CT (n = 37) and TT (n=26). *P* values were generated using Logrank test.

**Supplemental figure 4: Impact of NKp30 rs1052248 gene variants on expression of NKp30 and outcome of AML during immunotherapy.** **(A-D)** Median fluorescence intensity of NKp30 based on genotype status of NKp30 rs1052248 in AML patients in CD16^-^CD56^bright^ and CD16^+^ CD56^+^ NK cells before and after HDC/IL-2 therapy. Patients were dichotomized according to number of NKp30 rs1052248 T allele and numbers are 4, 16 and 41 before starting immunotherapy and 5, 19 and 38 after immunotherapy for no C allele, 1 C allele and 2 C alleles respectively. **(E, F)** Kaplan-Meier curves show impact of various NKp30 rs1052248 variants on leukemia-free survival and overall survival of AML patients after receiving HDC/IL-2 therapy. Simple linear regression analysis is performed to check how NKp30 rs1052248 gene variants can impact on NKp30 expression in different NK cell subsets during immunotherapy course in figures (A-D). Logrank test was used to study the survival based on NKp30 rs1052248 variants in figures (E-F).

|  | **Univariable cox** | | | **Multivariable cox** | | |
| --- | --- | --- | --- | --- | --- | --- |
| **Analysis** | **Hazard ratio** | **Confidence interval** | **p-value** | **Hazard ratio** | **Confidence interval** | **p-value** |
| DNAM-1 CD16+CD56+ MFI Before therapy, LFS | 2,731 | 1,309-5,700 | 0,007 | 2,46 | 1,169-5,18 | 0,018 |
| DNAM-1 CD16+CD56+ MFI Before therapy, OS | 1,838 | 0,697-4,844 | 0,218 | 1,56 | 0,585-4,157 | 0,374 |
| DNAM-1 CD16+CD56+ MFI induction C1D1 to C1D21, LFS | 0,428 | 0,199-0,921 | 0,03 | 0,458 | 0,212-0,988 | 0,047 |
| DNAM-1 CD16+CD56+ MFI induction C1D1 to C1D21, OS | 0,464 | 0,158-1,359 | 0,161 | 0,509 | 0,173-1,498 | 0,22 |

**Supplemental table 1: Multivariable analysis of age covariate on LFS**
